# An Interesting Case of Anti-Small Ubiquitin-Like Modifier-Activating Enzyme (SAE) Antibody-Positive Dermatomyositis

**DOI:** 10.7759/cureus.87833

**Published:** 2025-07-13

**Authors:** Smrithi Suresh, Suresh Kumar, Bhargavi M V, Srinivasan Ramadurai, Rajkumar Mani

**Affiliations:** 1 General Medicine, Sri Ramachandra Institute of Higher Education and Research, Chennai, IND; 2 General medicine, Sri Ramachandra Institute of Higher Education and Research, Chennai, IND

**Keywords:** antibodies, autoimmune disease, derrmatomyositis, dysphagia, muscle weakness, rituximab, steroid resistance

## Abstract

Dermatomyositis (DM) is a heterogeneous autoimmune disease characterized by inflammatory involvement of the skin and muscles. Anti-small ubiquitin-like modifier activating enzyme (SAE) antibodies define a rare clinical subtype of DM, often associated with prominent cutaneous findings and systemic features such as dysphagia. This report presents the case of a 45-year-old male with steroid-refractory, anti-SAE-positive DM, highlighting the diagnostic and therapeutic challenges of this condition and underscoring the importance of early recognition and personalized management.

## Introduction

Dermatomyositis (DM) is a type of idiopathic inflammatory myopathy (IIM) known for its hallmark skin rashes and muscle weakness. It is further classified into subtypes based on the presence of myositis-specific antibodies (MSAs) [1,2]. A large retrospective study conducted in Minnesota over 32 years estimated the incidence of DM at approximately 1 in 100,000 annually, with a prevalence of about 20 in 100,000 people [1].

Although anti-small ubiquitin-like modifier activating enzyme (SAE) antibodies are rare, they are increasingly recognized in patients who present with severe skin involvement and systemic complications such as dysphagia and interstitial lung disease (ILD). While many patients improve with corticosteroids, some do not respond, complicating management. This case illustrates the clinical course of a patient with refractory anti-SAE-positive DM and the challenges associated with its treatment.

## Case presentation

A 45-year-old man with a history of type 2 diabetes mellitus and dyslipidemia for six months (on Tab. Metformin and Tab. Atorvastatin), and hypertension for five years (on Tab. Telmisartan), presented with facial and body rashes for two months, along with progressive, symmetrical, proximal muscle weakness. This weakness led to difficulty in rising from a sitting position, raising his hands above his head, and recent onset of difficulty swallowing both solids and liquids, suggestive of oropharyngeal dysphagia. In the past two weeks, he also developed difficulty maintaining an upright head posture, indicating cervical extensor weakness. Notably, the patient had a history of unsupervised over-the-counter steroid use (Tab. Dexamethasone 4 mg once daily for 10 days, along with other undocumented drug intake). He had no prior history of infections or hospitalizations.

**Figure 1:**
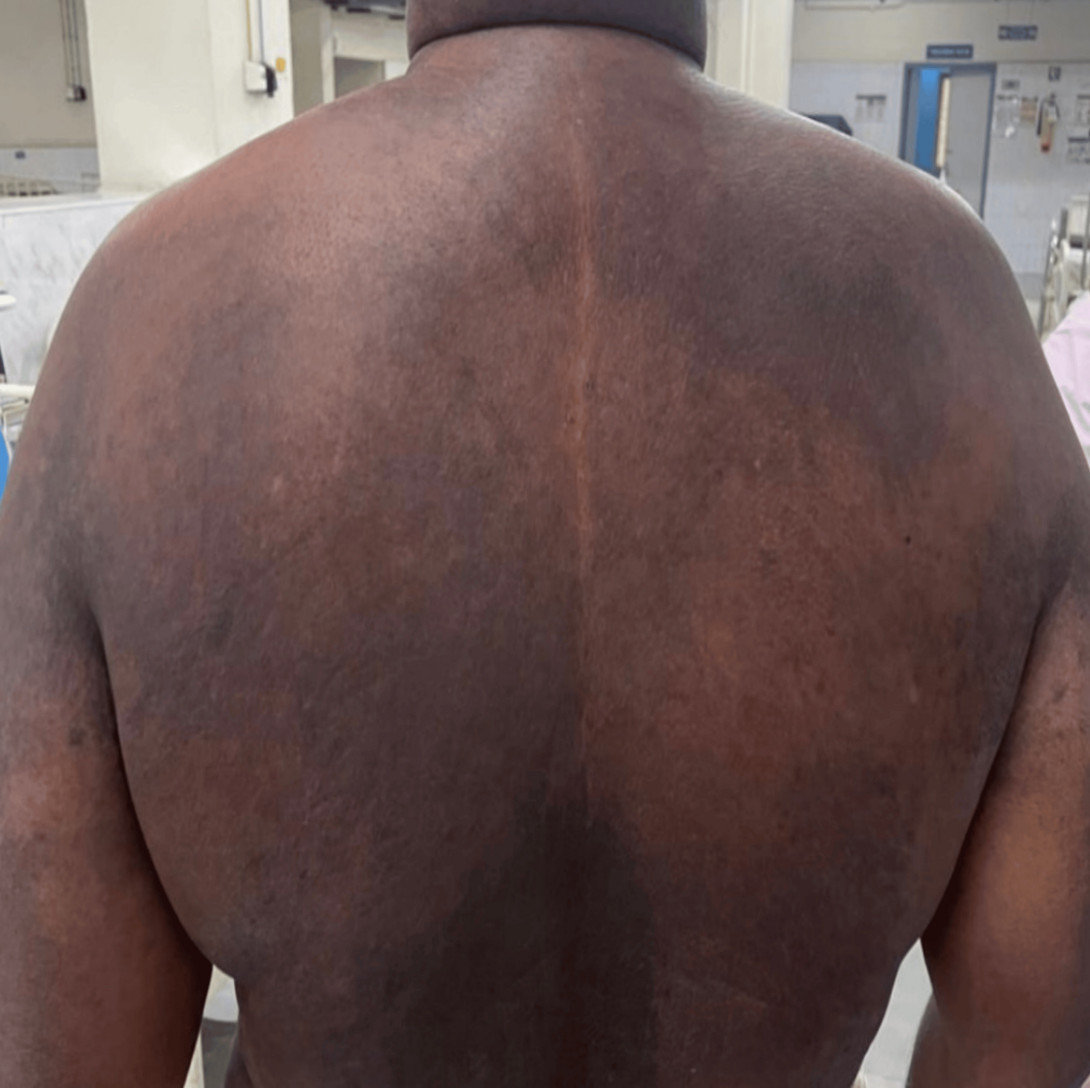
Angel wing sign: diffuse erythematous rash over the back.

**Figure 2:**
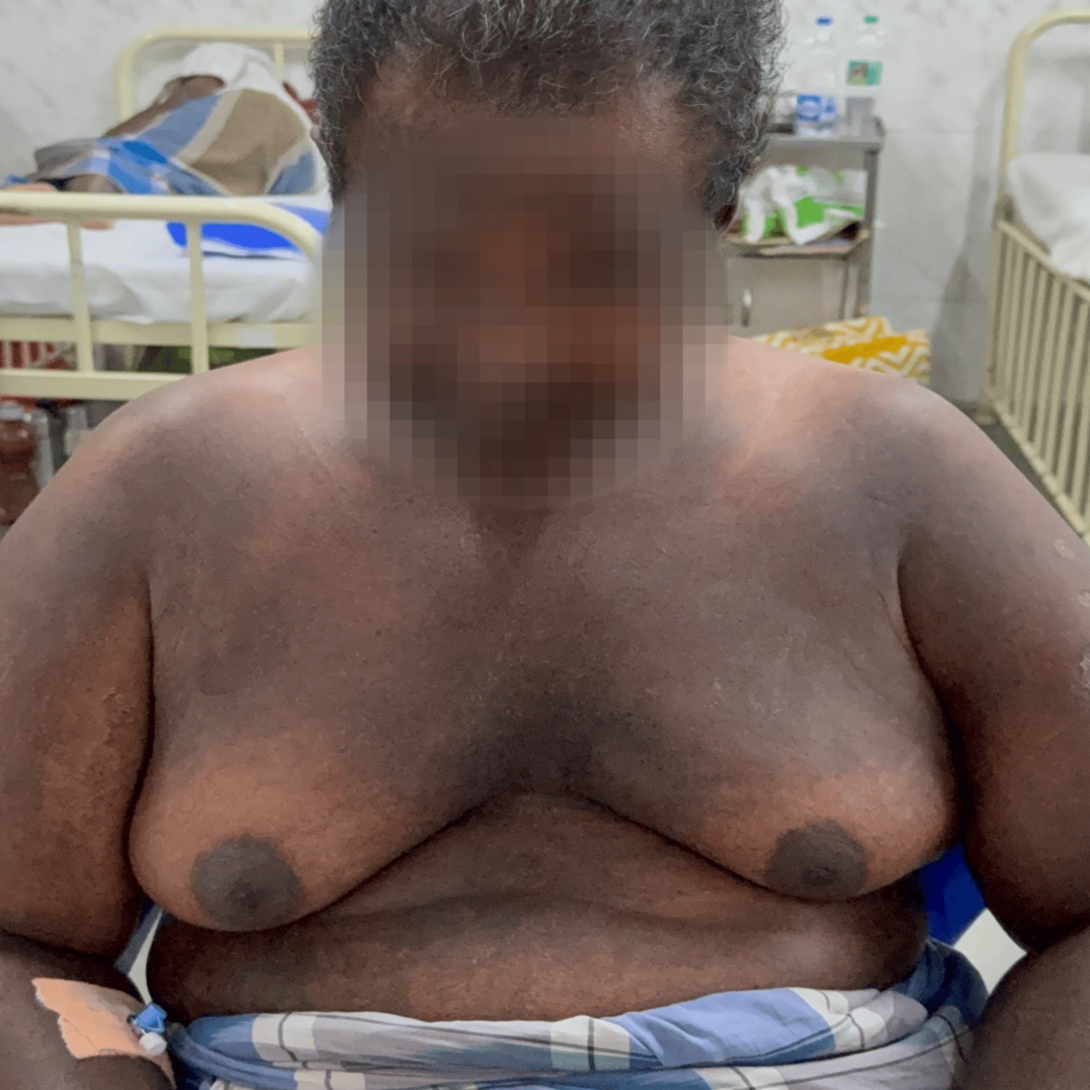
V sign: discoloration over the anterior chest and neck with peri-areolar sparing.

Examination revealed Gottron’s papules over bilateral metacarpophalangeal joints, periorbital edema with heliotrope rash, a diffuse erythematous rash over the back (Angel wing sign) (Figure 1), and violaceous discoloration over the anterior chest and upper neck with periareolar sparing (V sign) (Figure 2). Neurologically, he had symmetrical proximal muscle weakness (grade 4/5) with preserved reflexes and intact cranial nerves. Sensory examination was normal. Respiratory examination was unremarkable, except for a reduced single breath count of 16.

In view of the history of unsupervised steroid use, the patient was initially supplemented with Inj. Arachitol and calcium, and corticosteroids were withheld. Based on the clinical findings, myositis profile (line immunoassay (LIA) 23 panel) (Table 1), and muscle biopsy, a diagnosis of adult-onset dermatomyositis was established. Azathioprine 25 mg twice daily (later increased to 50 mg twice daily) and hydroxychloroquine (HCQ) were initiated along with supportive care. Despite treatment, the patient’s muscle strength deteriorated to grade 2/5, and his dysphagia worsened, necessitating corticosteroid therapy with Inj. Dexamethasone 10 mg/day along with antibiotics for 10 days, which resulted in transient improvement in muscle strength. Rituximab was planned; however, the patient developed metabolic acidosis and encephalopathy, leading to clinical deterioration and death.

**Table 1:** Laboratory evaluation. Hb: Hemoglobin; TLC: Total Leukocyte Count; SGOT: Serum Glutamic-Oxaloacetic Transaminase; AST: Aspartate Transaminase; SGPT: Serum Glutamic-Pyruvic Transaminase; ALT: Alanine Transaminase; ESR: Erythrocyte Sedimentation Rate; CPK: Creatine Phosphokinase; LDH: Lactate Dehydrogenase; ANA: Antinuclear Antibody; Anti-dsDNA: Anti-double-stranded DNA; SAE: Small Ubiquitin-like Modifier Activating Enzyme; LIA: Line Immunoassay; EMG: Electromyography.

Investigation	Patient Findings	Reference Range
Hemoglobin	11.1 g/dL	12-16 g/dL (female), 13.5-17.5 g/dL (male)
Total Leukocyte Count	5300/mm³ (Neutrophils 84%, Lymphocytes 7.5%)	4000-11,000/mm³ (Neutrophils 40-75%, Lymphocytes 20-45%)
Platelet Count	252,000/mm³	150,000-450,000/mm³
Serum Sodium	129 mmol/L	135-145 mmol/L
Serum Potassium	4.7 mmol/L	3.5-5.1 mmol/L
Serum Albumin	1.8 g/dL	3.5-5.0 g/dL
Serum Globulin	5.6 g/dL	2.0-3.5 g/dL
SGOT (AST)	217 IU/L	5-40 IU/L
SGPT (ALT)	53 IU/L	7-56 IU/L
Alkaline Phosphatase	248 IU/L	44-147 IU/L
ESR	Elevated	<20 mm/hr (varies by age and sex)
Creatine Phosphokinase (CPK)	Elevated (936 → 585 IU/L)	30-200 IU/L (lab-dependent)
Lactate Dehydrogenase (LDH)	452 IU/L	100-220 IU/L
ANA	Positive (1:320, speckled and nucleolar pattern)	Negative or <1:40
Anti-dsDNA	Positive	Negative
Myositis-Specific Antibodies	Positive for anti-SAE1 (80), anti-SAE2 (44)	Negative
Line Immunoassay (LIA-23) Panel	Positive for anti-PM/Scl75 (2+)	Negative
Muscle Biopsy	Lymphocytic infiltration (CD4+, CD8+, CD20+) in perivascular and endomysial regions	Normal muscle fibers without inflammation
Electromyography (EMG)	Nonspecific findings	-

## Discussion

DM presents with characteristic symmetrical proximal muscle weakness, often accompanied by cutaneous manifestations such as Gottron’s papules, heliotrope rash, and the “V” or “shawl” sign [1]. Systemic involvement, including dysphagia, dyspnea, and ILD, is not uncommon and contributes to morbidity and mortality in these patients [2].

Classification criteria for idiopathic inflammatory myopathies (IIM) have evolved from the early Bohan and Peter definitions to the more recent EULAR/ACR criteria (2017), which incorporate clinical features, serological markers, and muscle biopsy findings [3]. A score ≥5.5 (without biopsy) or ≥6.7 (with biopsy) is required for classification as IIM.

In our patient, the presence of classic cutaneous signs, dysphagia, elevated creatine phosphokinase (CPK), and positive anti-SAE antibodies fulfilled these criteria. Histopathology supported the diagnosis with perimysial and perivascular lymphocytic infiltration.

First-line therapy for DM includes corticosteroids combined with immunosuppressants such as azathioprine, methotrexate, or mycophenolate mofetil [4,5]. Refractory disease, defined as lack of response to these agents, requires escalation to intravenous immunoglobulin (IVIG), calcineurin inhibitors, or biologics like rituximab [6].

Anti-SAE antibodies: clinical significance 

Anti-SAE antibodies are found in about 8% of cases in Western populations [5]. They are associated with prominent skin involvement, delayed-onset muscle weakness, and a high incidence of systemic complications such as dysphagia and ILD. These patients often present with rash before muscular symptoms, a pattern observed in our case [7].

Dysphagia is a particularly important feature in anti-SAE-positive DM. It often compromises nutrition and increases the risk of aspiration pneumonia [7,8]. Steroid resistance may result from delayed diagnosis, older age, or previous unsupervised steroid use, which may alter immune responses.

The histological presence of CD20+ B cells supports the rationale for considering B-cell depletion therapy. Rituximab has shown benefit in steroid-refractory DM, especially in the presence of overlap syndromes and systemic features [8].

The different antibodies associated with DM and their manifestations are listed in Table 2.

**Table 2:** Antibodies commonly associated with dermatomyositis.

Antibody	Associated Features	Prognosis / Clinical Significance
Anti-Mi-2	Seen in classic dermatomyositis. Strong association with prominent skin findings like Gottron’s papules and heliotrope rash. Rarely seen in clinically amyopathic dermatomyositis (CADM).	Generally associated with a good prognosis. Patients respond well to treatment and have a lower risk of systemic complications.
Anti-MDA5 (Melanoma Differentiation-Associated Protein 5)	Commonly found in CADM. Strongly associated with rapidly progressive interstitial lung disease (RP-ILD), cutaneous ulceration, and palmar papules.	Poor prognosis due to risk of severe lung involvement. High mortality associated with RP-ILD, especially in East Asian populations.
Anti-TIF1-γ (Transcriptional Intermediary Factor 1-γ)	Frequently associated with cancer-associated dermatomyositis, especially in adult patients. Distinctive skin features include the V-sign and shawl sign. Rare in juvenile cases.	Significantly increased risk of underlying malignancy. Requires thorough and repeated cancer screening.
Anti-NXP-2 (Nuclear Matrix Protein 2)	Predominantly seen in juvenile dermatomyositis. In adults, associated with severe muscle weakness, dysphagia, and increased malignancy risk. May also present with calcinosis.	High risk of systemic complications and malignancy in adults. Requires careful monitoring and possibly aggressive treatment.
Anti-SAE (Small Ubiquitin-like Modifier Activating Enzyme)	Linked with amyopathic or hypomyopathic presentations. Patients often show systemic involvement and interstitial lung disease. May present initially with skin rash and delayed muscle involvement.	Risk of systemic disease, including ILD. Requires close clinical surveillance and monitoring for lung disease.
Anti-Jo-1	Most commonly associated with polymyositis but can be found in dermatomyositis. Strongly linked with interstitial lung disease, mechanic’s hands, arthritis, and Raynaud’s phenomenon.	Chronic disease course with frequent ILD. Treatment often includes immunosuppressive therapy. Requires pulmonary monitoring.

Therapeutic and pathophysiological considerations

Given the diverse clinical features of DM, early identification of antibody subtypes is crucial. Personalized therapy based on myositis-specific antibody (MSA) profiles and clinical severity is increasingly recommended [5]. Emerging therapies targeting SUMOylation pathways may prove effective for anti-SAE-positive disease [9,10]. Meanwhile, early multidisciplinary management of complications such as dysphagia remains critical for preventing adverse outcomes.

## Conclusions

Anti-SAE-positive DM is a rare but clinically significant subtype, often presenting with severe cutaneous manifestations, systemic complications, and potential treatment resistance. This case underscores the importance of early antibody testing, comprehensive evaluation, and timely escalation to advanced therapies such as rituximab in steroid-refractory disease. Further research into its pathophysiology and long-term outcomes is needed to guide targeted treatment strategies.
